# Numerical and experimental evaluation of the collapse resistance of MLPs under external pressure by lateral loading

**DOI:** 10.1038/s41598-026-47504-8

**Published:** 2026-04-28

**Authors:** Felipe Lutckmeier, Matheus Freitas Kuhn, Allan Romário de Paula Dias, Eduardo Becker Groth, Rodrigo do Nascimento Carvalhal, Håvar Ilstad, Thomas Gabriel Rosauro Clarke

**Affiliations:** 1 LAMEF – Physical Metallurgy Laboratory, Federal University of Rio Grande Do Sul (UFRGS), Av. Bento Gonçalves, 9500, Building 43820, Porto Alegre, Brazil; 2 Equinor, Equinor Research Center, Equinor Arkitekt Ebbells Veg 10, 7053 Trondheim, Ranheim, Norway; 3 Equinor, Equinor Brazil, Rua Do Russel, 804, Rio de Janeiro, Brazil

**Keywords:** Mechanically lined pipes, Finite element method, Experimental collapse testing, Engineering, Materials science

## Abstract

This study aims at evaluating the collapse resistance of mechanically lined pipes (MLPs) by comparing results from experimental and numerical methods. Initially the mechanical properties of the pipe materials, and the friction coefficient between the layers of MLP, were characterized through lab tests. This data was then used to develop numerical simulations, which were subsequently validated by comparing them to the results of full-scale collapse tests and the DNV-ST-F101 design equation. A parametric study was performed to assess the influence of the friction coefficient and the thickness of the liner on collapse pressure. The findings indicate that the CRA liner provides significant structural reinforcement, leading to collapse pressures that exceed standard design predictions, particularly for MLPs with thick liners. Furthermore, the interfacial friction was also shown to be a contributing factor to this enhanced resistance.

**Supplementary Information:**

The online version contains supplementary material available at 10.1038/s41598-026-47504-8.

## Introduction

Subsea pipelines are crucial for fluid transportation from subsea wellheads to surface production facilities in off-shore field developments^1–3^. In this scenario, mechanically lined pipes (MLPs) have emerged as a cost-effective solution to overcome the issues caused by the action of corrosive compounds on plain carbon steel pipes^4–6^. Although design codes such as DNV-ST-F101^7^ acknowledge that the corrosion-resistant alloy (CRA) layer in MLPs can influence the overall mechanical properties of pipes, the extent of these effects is not fully documented or understood. One of the potential causes of failure in these pipelines is collapse^8^. Pipeline collapse involves a loss of stiffness that compromises the structural integrity of the pipe, leading to severe cross-sectional deformation, such as ovalization, or a complete collapse if the external pressure is not relieved. This is a critical failure and it takes place when the pipeline exceeds a pressure limit defined as the “collapse pressure” (P_C_)^9^. The collapse pressure of a pipeline depends on a number of external variables, such as the level of pipe ovalization, pressure loading, wall thickness, stress–strain response, and residual stresses due to the manufacturing process. In addition, for thick-walled pipelines, where collapse is governed by plastic yielding, the kinematic strain-hardening effect (Bauschinger effect) becomes highly relevant, as it directly affects the compressive strength of the material^10–12^. The effect of the CRA layer on the collapse properties of MLPs has been investigated through numerical simulations^13–15^ with the aim of reducing the conservatism of design codes; however, only a limited number of papers have experimentally validated this observation^15^. In particular, few papers investigate the role of geometrical and physical properties on the collapse resistance of MLP by adopting a methodology linking experimental measurements to meso/micro-scale mechanisms and presenting damage evolution quantitatively such as in^16^.

This study combines full‑scale external‑pressure collapse testing of mechanically lined pipes (MLPs) with a comprehensively calibrated three‑dimensional finite element framework, providing a detailed characterization of liner–steel interaction mechanisms under realistic loading conditions. The experimental program covers a broad range of backing steel diameter-to-wall thickness ratios and liner thicknesses, offering data that complement previous numerical or small‑scale investigations. In parallel, interfacial friction between the CRA liner and backing steel is measured directly through an adapted ASTM G115‑10^17^ procedure and incorporated into the finite element contact formulation, enabling a consistent evaluation of composite action. The study also establishes a transparent comparison against the collapse expressions in DNV‑ST‑F101, examines the role of geometric and material variability, and synthesizes the parametric trends associated with liner diameter-to-wall thickness ratio, ovality, and friction. Together, these elements form a unified experimental–numerical basis for interpreting the collapse response of MLPs subjected to external pressure and for supporting physics‑based design considerations.

## Experimental full-scale pipe collapse tests

This investigation evaluates the collapse resistance of 21 mechanically lined pipe specimens, each comprising a backing steel pipe of API 5L X65 and an Inconel 625 liner, through comprehensive numerical and experimental analysis. To facilitate the examination of a broad spectrum of diameter-to-thickness ratios for the backing steel (D_BS_/t_BS_), spanning from 8.9 to 21.2, the external diameter of select specimens was systematically reduced via machining. All specimens possessed a length of 3 m and were configured with corrosion resistant alloy (CRA) liners with nominal thicknesses of 3 mm or 4 mm.

Previous studies suggest that the application of thermal insulation coating to MLPs can cause redistribution or relief of residual stresses. This could impact the gripping force in the pipes^18^, as well as triggering metallurgical effects that can alter their mechanical properties^19,20^. To simulate the application of this insulating coating, all specimens were subjected to a heat treatment at 250 °C for 900 s.

The pipe ovality of the specimens was estimated by the pipe diameter variations measured at different circumferential positions using a caliper gauge. The wall thickness of the outer-pipe was mapped by conventional ultrasonics through the pulse-echo method, and the measurements were used to determine the percentual pipe eccentricity in the carbon steel pipes. The measurements were taken every 0.5 m along the length of the pipe and every 30° in the hoop direction. In addition, material samples of the carbon steel and Inconel alloy were extracted from the pipe segments for uniaxial tensile tests, according to ASTM E-8 standard^21^.

**Fig. 1:**
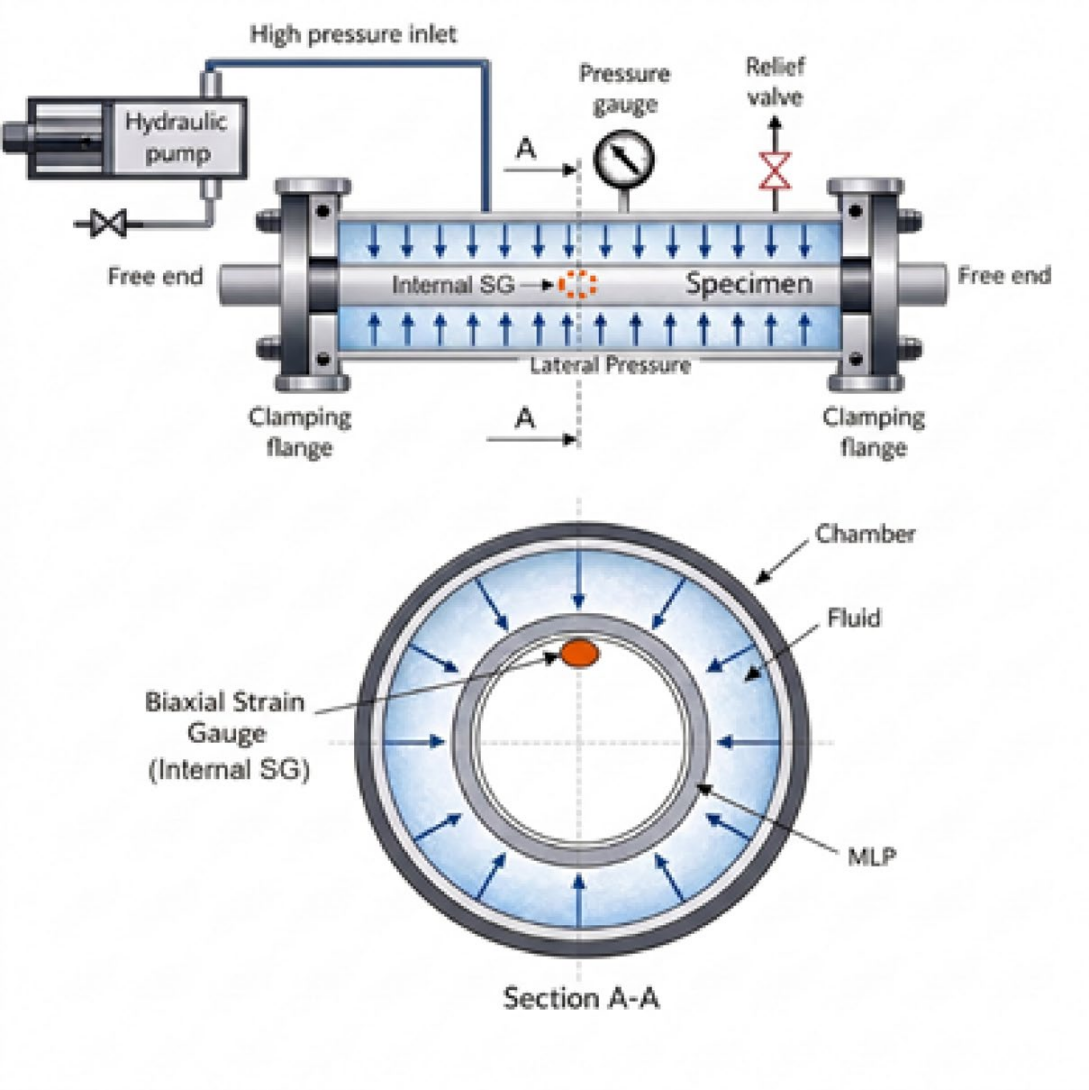
Schematic of the pressure chamber used in full-scale collapse testing.

Collapse tests were conducted in a lateral loading pressure chamber at the Vallourec Soluções Tubulares do Brasil facilities. In contrast to hydrostatic conditions, this method applies pressure exclusively to the lateral surface of the pipe, allowing the pipe ends to displace freely in the axial direction. Lateral loading is generally considered to produce more conservative results than hydrostatic loading since it leads to lower values of collapse pressure of pipes^10,15^. A pressure zone extending over 2.6 m ensured that the length of the pipe subjected to loading exceeded ten times the outer diameter (10 times outer diameter (OD)) of the pipe. The load bearing area was at least 1 OD away from the edges to prevent end-effects from influencing the measurements, and pressure containment was secured by clamping flanges with elastomeric seals at these positions. The chamber, shown schematically in Fig. 1, has a maximum pressure capacity of 140 MPa. As shown in the figure, a biaxial strain gauge was attached to the internal surface of the pipe at the mid-length of the specimen through specially developed tools in order to capture the strain up to pipe collapse.

**Fig. 2:**
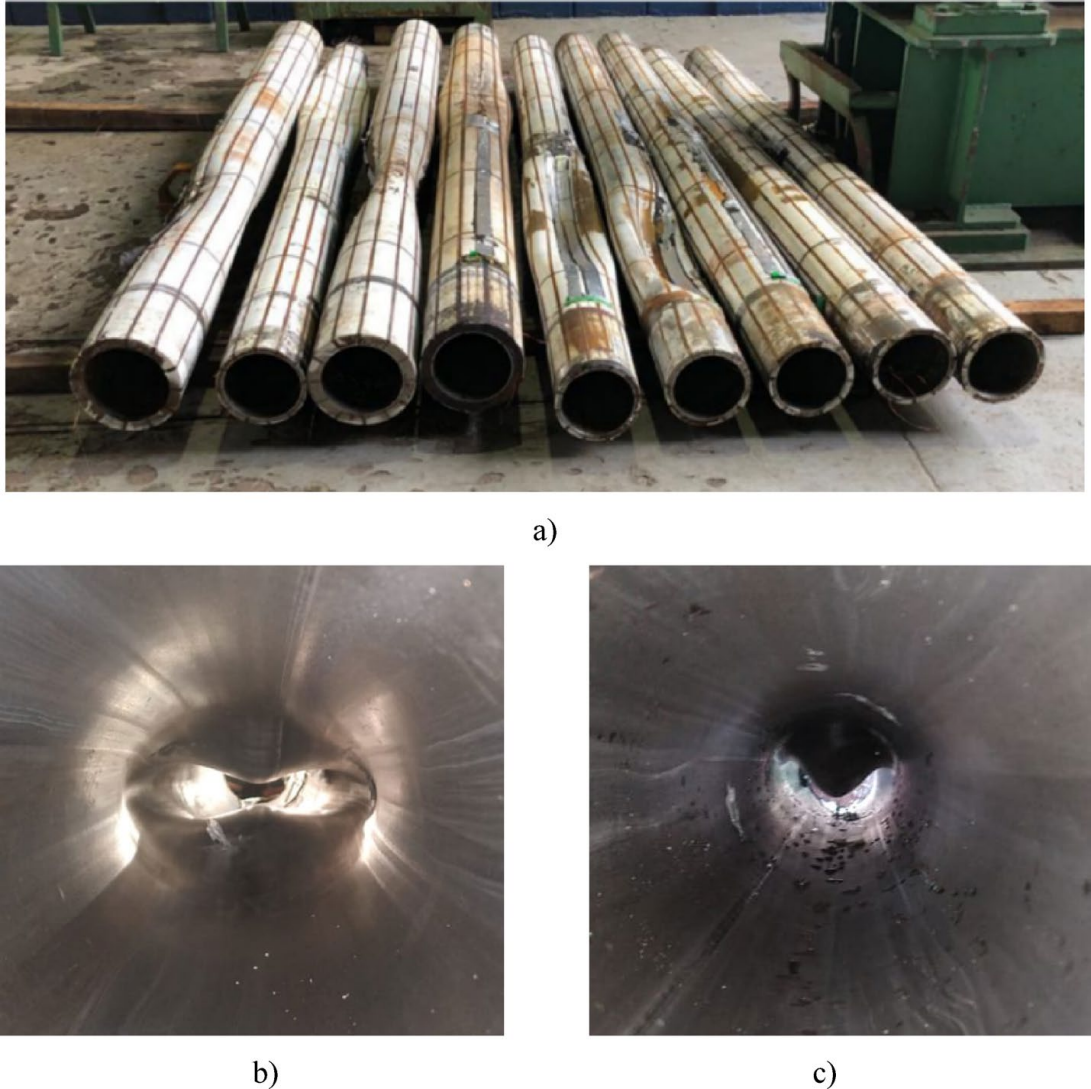
(**a**) Specimens after collapse test. (**b**) Dog-bone mode buckled pipe. (**c**) U-shape mode buckled pipe. Adapted from^15^.

During the collapse tests, some specimens collapsed into a “U-shape” and some collapsed into a “dog-bone shape”, as illustrated in Fig. 2. Supplementary Table S1 shows the collapse mode for each pipe. The observed variations in the collapse shape are attributed to differences in pipe wall thickness, pipe eccentricity, and material fluctuations^15^. It should be noted that the machining process used to achieve the target wall thickness produced eccentricity values that were somewhat higher than those typically observed in standard pipe specimens. Given its pronounced level in the tested specimens, this machining-induced eccentricity is therefore a primary suspect for the divergence in collapse modes observed in this study. As can be seen in Table S1, there are a number of specimens in which high pipe eccentricity values led to U-shape failures, with a few exceptions that are likely due to a combination of geometrical effects.

## Friction measurements

The mechanical interaction of the liner with the surrounding steel backing in MLP is still not completely understood, but previous studies suggest that these effects can influence the collapse performance of multilayered pipes^22^. This phenomenon—often described as the gripping force—arises from contact and friction behaviour between the layers^23–25^, as well as from residual stresses introduced during manufacturing by hydroforming (where both pipes are expanded using internal hydraulic pressure). Thermal cycles imposed during subsequent manufacturing steps, reeling and installation operations^26,27^, can also modify the stress state and contact characteristics. Measuring the contribution of residual stresses to the gripping force is challenging since sectioning of the pipe leads to significant relief and redistribution, and only advanced and expensive techniques, such as neutron diffraction, may, under certain conditions, allow in-situ measurements in full-scale pipes samples. Friction measurements, on the other hand, can be more readily accomplished, and will be the focus of this work.

The procedure used to determine the friction coefficient between the carbon steel pipe and the CRA liner is an adaptation of the ASTM G115-10 standard^17^ with details presented by Echer et al.^28^. This standard covers the determination of the friction coefficient through the sliding of two materials against each other under specific test conditions. For this test, one sample of the CRA liner (140 mm long and 55 mm wide) and one sample from the backing steel (200 mm long and 58 mm wide) were removed from twelve different pipes in the longitudinal direction. Samples were therefore slightly curved due to the circumference of the pipe. A steel wire cable was attached at one end to the CRA liner sheet and at the other end was connected to the fixing device of a universal testing machine (load cell), passing through a pulley. The backing steel sample is considered a stationary base. A load is applied to the tensile machine until the CRA liner sheet slides over the backing steel base. Dry friction occurs at the interface when relative motion is introduced into the system. The friction force opposes the direction of the relative velocity and depends on the normal force acting on the body. The friction force is proportional to the normal force, with the proportionality constant being the friction coefficient, as shown in Eq. 1:

1 $${F}_{FRIC}= {\upmu }_{S} . {F}_{N}$$

where m_s_ is the static friction coefficient, F_FRIC_ is the maximum force before sliding, and F_N_ is the normal force acting on the body. Standard weight blocks were placed on the study material to provide a known normal force, using three configurations that resulted in approximate total weights of 18.5 kg, 32.0 kg, and 45.7 kg. Each CRA/steel pair was tested three times for each load, totaling nine measurements for each of the twelve samples. The static friction coefficient was then calculated according to Eq. 2:

2 $${\mu }_{s}=\frac{{F}_{FRIC}}{\left({m}_{CRA}+{m}_{add}\right)\bullet g}$$

Here, m_CRA_ is the CRA sample mass, m_add_ the added weight, and g is gravity (9.81 m/s^2^). The total number of measurements was 108, leading to a distribution that was approximately Gaussian, with mean value of 0.45 and a standard deviation of 0.03.

## Finite element modelling (FEM)

**Fig. 3:**
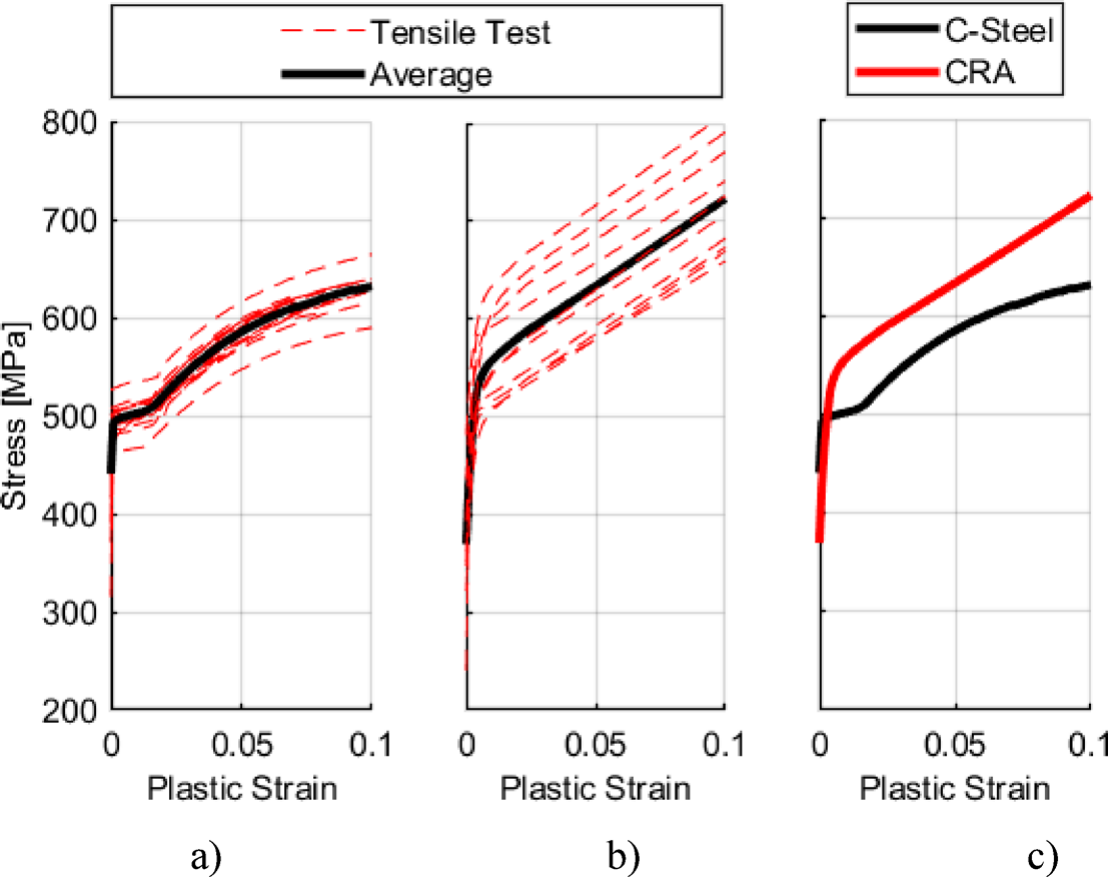
Finite element modelling material; (**a**) Stress–Strain curves for C-Steel; (**b**) Stress–Strain curves for CRA Liner; (**c**) Mean Stress–Strain curves for C-Steel and CRA Liner.

Finite element models were developed to reproduce the loading and boundary conditions of full-scale tests, considering the specific geometric parameters and material properties of MLPs. Also, a parametric study was conducted to populate a database, aiming to evaluate the impact of the diameter to wall thickness ratio of the CRA liner (D_CRA_/t_CRA_) and the friction coefficient on the collapse pressure. In models and experiments the parameters D_CRA_ and t_CRA_ refer to the nominal outer diameter and wall thickness of the liner, respectively. The D_CRA_/t_CRA_ ratio expresses the slenderness of the liner, and it will be adopted to describe the relationship between the liner and the backing steel pipe (which is usually represented by its nominal outer diameter to nominal wall thickness ratio D_BS_/t_BS_) during collapse. The analyzed parameters and their values are listed in Table 1. For the simulations, the average stress–strain curves obtained from experimental tests for each material were used. Figure 3a presents the curves for carbon steel, Fig. 3b for CRA liner, and Fig. 3c provides a comparative view of both.

**Table 1:** FEM parameters.

Parameter	Values
Outer Diameter—D_BS_	225 mm
Length	5 × D_BS_ = 1125 mm
Diameter-to-thickness ratio of the backing steel (D_BS_/t_BS_)	7.5 to 22.5, in increments of 2.5
Diameter-to-thickness ratio of the CRA liner (D_CRA_/t_CRA_)	20 to 90, in increments of 2.0
Ovality—*f*_0_	0.1% and 1.0%
Static Friction Coefficient—µ_S_	0 and 0.45
Eccentricity—E_CC_	0

The design code DNV-ST-F101^7^ provides the equations for calculating ovality (f_0_) and eccentricity (E_CC_) of pipes, which are presented in Eqs. 3 and 4. Where D_max_, D_min_, and D_BS_ are the maximum, minimum, and average backing steel diameter, and t_max,_ t_min_, and t_BS_ are the maximum, minimum, and average backing steel wall thicknesses, respectively.

3 $${f}_{0}=\frac{{D}_{max}- {D}_{min}}{{D}_{BS}}$$

4 $${E}_{CC}=\frac{{t}_{max}- {t}_{min}}{{t}_{BS}}$$

The initial wall-thickness eccentricity was not included in the numerical models, and a uniform-thickness cross-section was assumed instead. This simplification was adopted to enable the use of geometric and loading symmetry conditions, which allows modelling only a fraction of the pipe’s cross-section, thereby reducing computational cost and permitting greater mesh refinement. Results presented in^29^ show that, for eccentricity levels of approximately 10%, the reduction in collapse resistance is secondary compared to the effect of initial ovalization. According to the authors, within this range of values, ovalization remains the governing parameter, with the reduction in collapse resistance due to eccentricity being less than 5%.

**Fig. 4:**
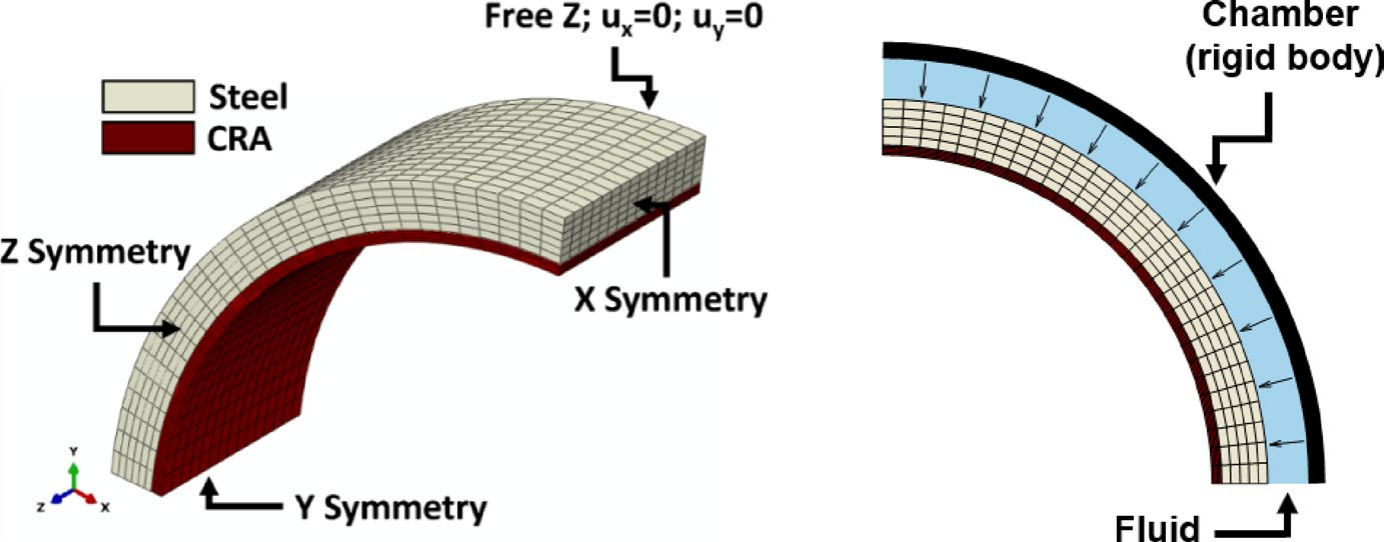
FEM model showing the pipe and boundary conditions. Dimensions of elements are not to scale for better visualization.

A dynamic implicit solution algorithm was used in combination with a fluid-cavity interaction model in Abaqus to simulate the collapse problem. This solution method works by introducing a mass flux to the predefined fluid cavity which is represented by generated hydrostatic fluid elements. The mass flux gradually increases the hydrostatic pressure in the fluid cavity until it reaches the collapse capacity of the MLP. The post-peak behavior is then characterized by the MLP deforming sufficiently fast for the pressure in the fluid cavity to drop in a controlled manner. This allows for a converging solution that is able to determine the peak response of a classical “snap-through problem”. The solid geometries (CRA liner and steel pipe) were modeled using C3D8R linear integration elements, while the chamber fluid was represented by F3D4 elements. The contact between the steel pipe and the CRA liner was characterized by two terms: a tangential behavior with or without a penalty (which is the measured static friction coefficient) to consider or not the effect of friction, and a normal rigid contact behavior. After contact, the surfaces were free to separate. The chamber was treated as a rigid body. The clamps of the pressure chamber were modeled by imposing constraints on the displacements in the x and y axes (uₓ = 0 and uᵧ = 0), while the displacement in the z axis was left free. These boundary conditions replicate faithfully the conditions found during testing, thus minimizing their contribution to differences found in comparisons between experimental and modelling results. All applied boundary conditions are illustrated in Fig. 4. As mentioned, eccentricity was not considered in the model, and symmetry planes were adopted in the x, y, and z axes, resulting in a simplified model representing 1/8 of the complete pipe.

A mesh convergence study was conducted to determine the appropriate number of elements in the longitudinal, circumferential, and thickness directions of the solid. Different quantities of elements were tested: in the thickness (2, 3, 4, 5, 6, and 7 elements), in the circumferential direction (20, 25, 30, 35, 40, and 45 elements), and in the longitudinal direction (20, 30, 40, 45, and 50 elements). Through an iterative process, by adopting a convergence criterion based on a relative variation in collapse pressure lower than 1% between successive refinements, we established as appropriate having 6 elements in the thickness, 40 elements in the circumferential direction, and 45 elements in the longitudinal direction for both materials, C-Steel and CRA Liner. Supplementary Table S2 in the Supplementary Information shows a summary of the convergence study that was performed.

## Results for experimental testing and FEM

Section 5.4.3 of the DNV-ST-F101 standard^7^ gives the characteristic collapse pressure (P_C,DNV_) of a plain steel pipe as:

5 $$\left({P}_{C,DNV}-{P}_{el}\right).\left({{P}_{C,DNV}}^{2}-{{P}_{pl}}^{2}\right)={P}_{C,DNV}.{P}_{el}.{P}_{pl}.{f}_{0}.\frac{{D}_{BS}}{{t}_{BS}}$$

where ${D}_{BS}$ is the outer diameter and ${t}_{BS}$ is the wall thickness of the backing steel pipe, P_el_ is the elastic collapse pressure, and P_pl_ is the plastic collapse pressure of the pipe; the latter two are calculated according to Eqs. 6 and 7, respectively:

6 $${P}_{el}= \frac{2.E}{(1-{\nu }^{2})}.{\left[\frac{{t}_{BS}}{{D}_{BS}}\right]}^{3}$$

7 $${P}_{pl}= {\alpha }_{fab}.2.{\sigma }_{y}.\left[\frac{{t}_{BS}}{{D}_{BS}}\right]$$

where $E$ is Young’s modulus, $\nu$ is Poisson’s ratio, and ${\sigma }_{y}$ is the yield strength of the backing steel; ${\alpha }_{fab}$ is a manufacturing factor (Sect. 5.3.3), which was considered to be 1.0 for seamless pipes as are the ones considered in this work.

**Fig. 5:**
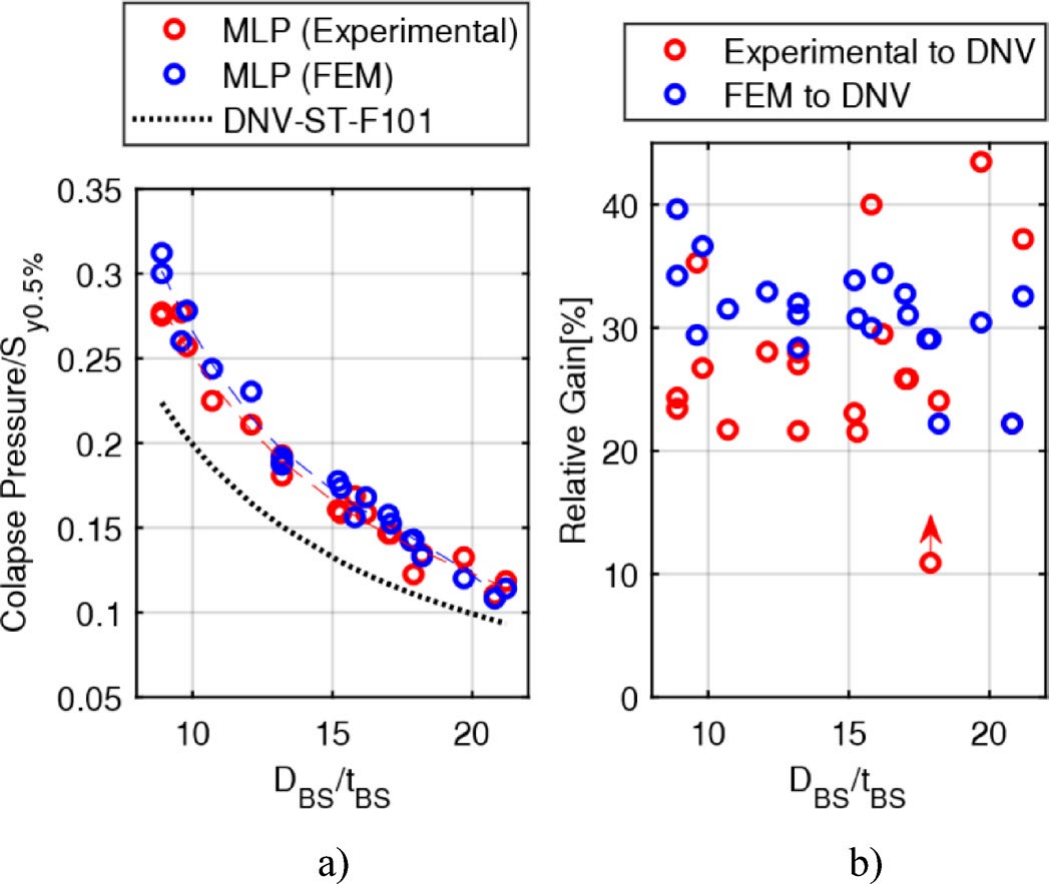
Comparison between experimental, FEM, and DNV results. (**a**) Ratio of collapse pressure to yield stress as a function of D_BS_/t_BS_; (**b**) Relative gain compared to DNV for both experimental and numerical results.

As illustrated in Fig. 5, both the experimental and numerical analyses yielded collapse pressures that significantly exceed the predictions from the DNV design equation, which was calculated without safety factors. This is because DNV-ST-F101 is intended for single-layer pipes; therefore, it underpredicts MLP collapse resistance as it fails to capture multi‑layer interaction, friction, or composite action. Nevertheless, it is used here as a baseline value for comparisons. Figure 5 shows that the magnitude of the improvement, or gain, of the lined pipe relative to the DNV prediction is also dependent on the diameter-to-thickness ratio of the pipe (D_BS_/t_BS_).

The numerical simulations presented in Fig. 5 were performed using the measured parameters of each individual specimen. All experimental data, specimen dimensions, and simulation results given in this section are listed in Table S1 in the Supplementary Information.

The main sources of uncertainty leading to differences in the comparisons between FEM and experimental results were variations in the pipe wall thickness and fluctuations in material properties. These issues were dealt with by using the average measured wall thickness for each pipe, and the mean stress–strain curves shown in Fig. 3. The eccentricity generated by the machining process adopted to increase the range of D_BS_/t_BS_ available, which is not considered in the models, is also thought to contribute to the differences, although, as mentioned earlier, this effect is smaller compared to ovalization (which is considered in the models).

The discrepancy in Fig. 5 is particularly pronounced for thicker pipes, with gains reaching 43%. This occurs because the DNV equation does not fully account for effects such as triaxial stress nor strain hardening that take place in thicker-walled pipes^30^. One data point (marked with a vertical arrow in the figure), is an exception, showing a gain of less than 20%. This result is considered an experimental anomaly since a leak in the chamber was detected during final pressurization, which likely induced premature ovalization and reduced the measured collapse resistance when the test was resumed.

**Fig. 6:**
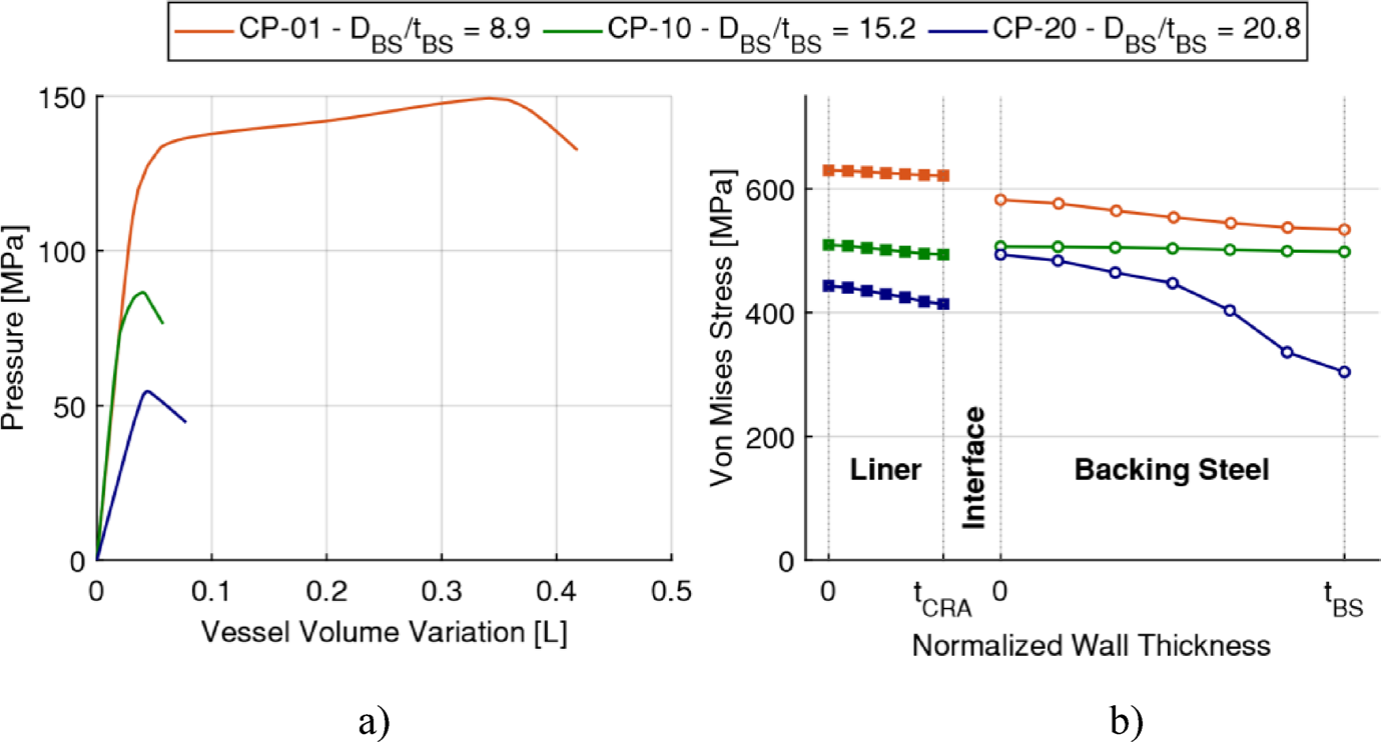
(**a**) Pressure versus volume variation curves from the FEM models for CP_01, CP_10 and CP_20. (**b**) Through-thickness von Mises stress distributions at the critical collapse state for each configuration.

Figure 6a illustrates the pressure curves as a function of the variation in pressurized fluid volume for specimens CP_01, CP_10, and CP_20, whose D_BS_/t_BS_ ratios are 8.9, 15.2, and 20.8, respectively. It is observed that CP_01 exhibits a typical plastic collapse behavior, characterized by significant permanent deformation prior to the loss of structural stability. CP_10 shows an elasto-plastic collapse response, presenting incipient plastic deformation preceding failure. Finally, CP_20 represents a case of elastic collapse, in which failure occurs due to geometric instability while the material is still in a strictly elastic regime.

Figure 6b presents the distribution of von Mises stresses along the cross-section of the pipes at the instant of maximum pressure for each scenario. To allow for a direct comparison between the different geometries, the thicknesses of the inner liner and the outer backing steel were normalized.

In specimen CP_01, a typical behavior of thick-walled cylinders under external pressure is observed, characterized by a stress gradient decreasing toward the outer surface. In this regime, the liner concentrates the highest levels of equivalent stress, reaching generalized yielding, while the backing steel provides high radial and circumferential stiffness, limiting ovalization and promoting the global stability of the section.

As slenderness increases, a progressive transition in the governing resistance mechanism becomes evident. In CP_10, the stress redistribution resulting from the interaction between material and geometric nonlinearities leads to an approximately uniform stress field across the layers, characterizing an elasto-plastic collapse regime in which yielding and instability simultaneously contribute to the loss of load-carrying capacity.

For CP_20, a significant change in the equivalent stress profile is observed at the critical instant, with stresses predominantly concentrated in the backing steel. This behavior is associated with the increased sensitivity to geometric instability, which induces pronounced ovalization and generates additional bending components in the backing steel. Consequently, collapse occurs predominantly by elastic instability before generalized yielding of the material develops.

Pipes with intermediate slenderness ratios (D_BS_/t_BS_ ≈ 15–20) exhibit a mixed collapse response in which neither yielding nor geometric instability dominates exclusively. In this range, the deformation process begins with ovalization, but localized yielding in both the liner and backing steel develops before geometric collapse is fully established. This interaction produces stress distributions that are more uniform than in thick‑walled specimens, yet less instability‑driven than in slender pipes, as seen in Fig. 6. The pressure–volume curves in this regime, such as that of CP_10, reflect this dual behavior: the response lacks the extensive plastic plateau of thick pipes and the abrupt elastic snap-through seen in slender pipes. The transitional range is also the most sensitive to variations in ovality, liner thickness, and friction, since these parameters influence whether yielding or geometric instability becomes the dominant mechanism. As a result, the collapse mode in this interval can shift between dog‑bone and U‑shape depending on small geometric or material variations.

Overall, specimens with D_BS_/t_BS_ between roughly 15 and 20 represent a mechanically hybrid region where the contribution of the liner and the effects of imperfections are maximized, making this range especially important for accurate modelling and design.

**Fig. 7:**
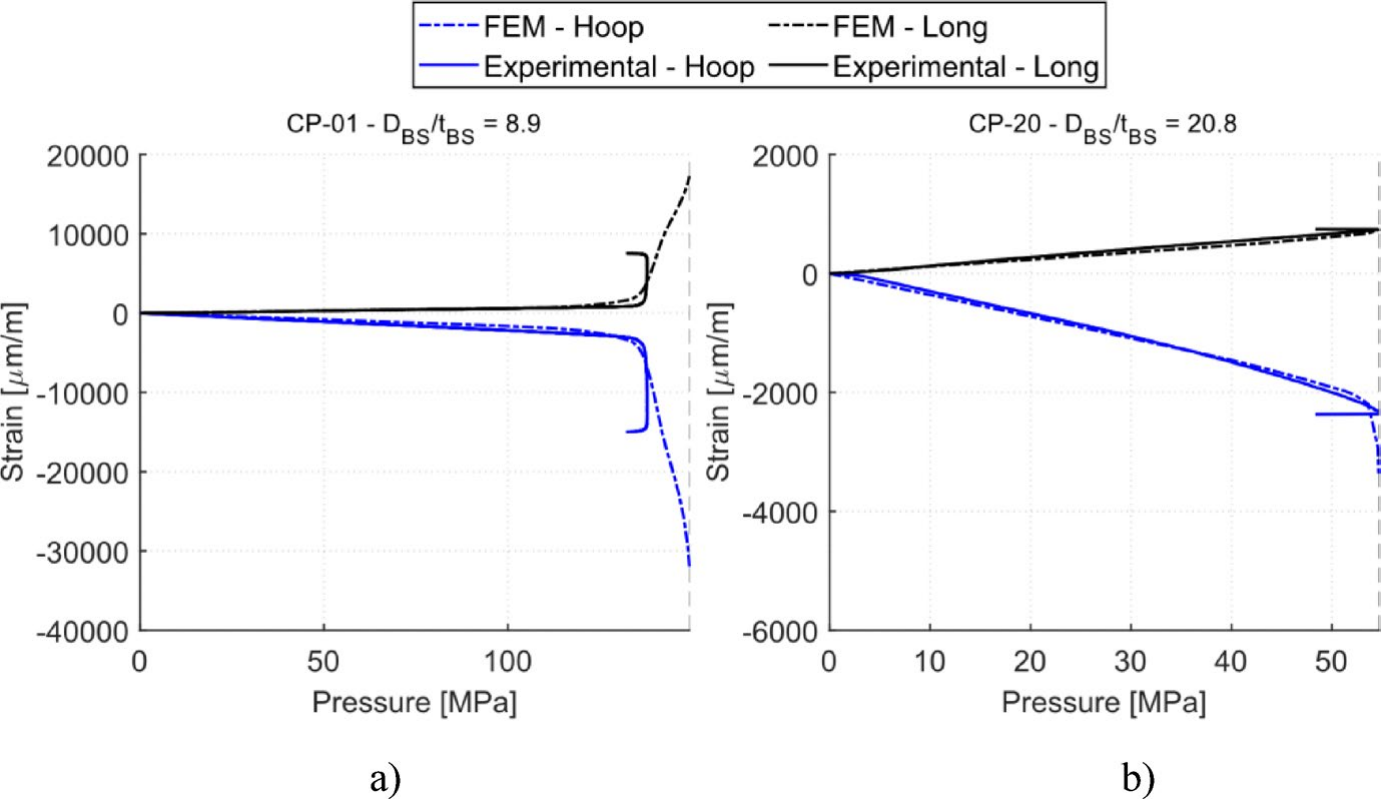
Comparison between experimental and modelled strain results for: (**a**) CP-01, D_BS_/t_BS_ = 8.9. (**b**) CP-20, D_BS_/t_BS_ = 20.8.

Figure 7 presents the correlation between numerical and experimental results for longitudinal and circumferential strains for specimens CP‑01 and CP‑20 at the strain-gauge location indicated in Fig. 1. A good agreement is observed in the elastic regime for both samples. This consistency in the initial slopes of the curves demonstrates the ability of the numerical model to represent the stiffness of the lined pipe (MLP) before the onset of severe plastic or geometric instabilities.

In Fig. 7a slight offset is observed between the experimental and numerical data at the beginning of loading. This phenomenon arises from the fact that the numerical model assumes perfect bonding and ideal contact between the liner and the backing steel from the initial state. In the physical system, the presence of gaps or mechanical clearances causes a delay in the experimental strain response until external pressure promotes full contact between the layers. The strain behavior in CP‑01 (D_BS_/t_BS_ ratio = 8.9) characterizes a plastic collapse scenario, sustaining significantly high pressures that drive the material into deep plastic deformation prior to total failure. In this case, a greater difficulty in modeling severe plastification within the plastic zone is observed, which is expected due to the highly nonlinear and complex nature of plastic instability phenomena in thick‑walled tubes.

Specimen CP_20, with a D_BS_/t_BS_ ratio of 20.8, exhibits a typical elastic collapse behavior (Fig. 7b). From the experimental results, the instability occurs abruptly at low strain levels, indicating that the loss of geometric stability precedes any significant material plastification. The numerical results show slight plastification prior to collapse, which may be associated with simplifications of the numerical model.

**Fig. 8:**
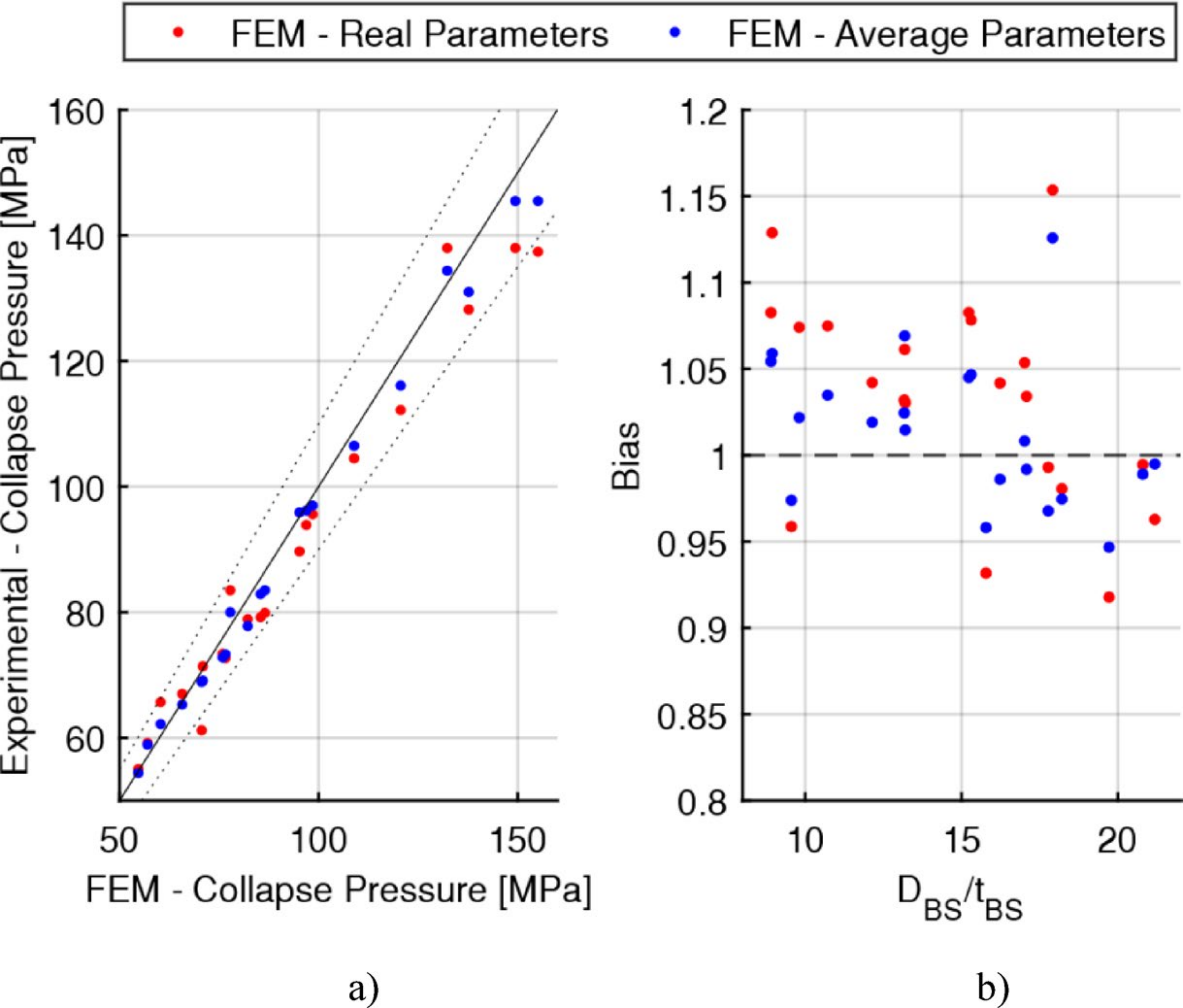
(**a**) Correlation between experimental results and finite element analysis. (**b**) Bias values obtained from Eq. 9.

Figure 8a presents, on the horizontal axis, the collapse pressures obtained from numerical simulations, using two sets of input parameters: the measured geometry and material properties of each specimen, and the average values derived from the full dataset. In both cases, the computational models consider the boundary conditions and loading characteristics of a full-scale collapse test. The vertical axis represents the collapse pressure measured from the experiments. Each point on the graph represents a pair of results for a specific specimen, comparing the experimental and numerical collapse pressure values. The identity line x = y represents the ideal condition where the collapse pressure calculated by the numerical model coincides with the experimentally obtained collapse pressure. The error bands indicated by the dashed lines confine the data within a ± 10% range. Most of the points are concentrated near the line x = y, indicating a strong positive linear correlation between the numerically calculated and experimentally obtained collapse pressures. The numerical simulations performed using the individually measured parameters for each specimen yielded a Pearson correlation coefficient^31^ of 0.983, as defined in Eq. 8, where r is the Pearson correlation coefficient, n is the number of specimens, P_C,FEM_ is the numerical collapse pressure, P_C,EXP_ is the experimental collapse pressure, and P̅_C,FEM_ and P̅_C,EXP_ are the average values of the numerical and experimental collapse pressures, respectively. In contrast, the simulations based on average parameter values resulted in a Pearson correlation coefficient of 0.993.

8 $$r = \frac{{\sum }_{i=1}^{n}\left({P}_{C,FEM,i} - {\overline{P} }_{C,FEM}\right)\left({P}_{C,EXP,i} - {\overline{P} }_{C,EXP}\right)}{\sqrt{{{\sum }_{i=1}^{n}\left({P}_{C,FEM,i} - {\overline{P} }_{C,FEM}\right)}^{2}}\sqrt{{{\sum }_{i=1}^{n}\left({P}_{C,EXP,i} - {\overline{P} }_{C,EXP}\right)}^{2}}}$$

For a clearer visualization of the results, Fig. 8b presents a comparison of numerical and experimental values as a function of D_BS_/t_BS_, with the bias values calculated using Eq. 9. A literature review did not identify studies directly correlating the collapse pressure obtained from numerical and experimental analyses of MLPs in full scale tests. However, investigations conducted by Xu et al*.*^32^ and Moreira Junior et al*.*^33^ on linerless pipes have evaluated this correlation identifying differences between numerical and experimental results ranging from 0.11% to 14%. Yuan et al.^34^ assessed both numerical and experimental collapse pressures for small-scale MLPs and observed a maximum discrepancy of 5.29% between the two sets of data. Considering these findings, it can be suggested that the developed numerical models are well calibrated and are capable of accurately estimating the collapse pressure of an MLP. Two specimens presented results outside the ± 10% error margin, with differences of 12.9% and 15.4%. The specimen that exhibited a 15.4% error was the one in which a leak occurred during pressurization.

Both sets of numerical results show a strong correlation with the experimental data, as evidenced by the high Pearson correlation coefficients in each case. This confirms the reliability of the computational in simulating the collapse behavior of the specimens, whether using individually measured or average parameters. For consistency and simplicity, the remaining simulations in this study were conducted using the average parameter values.

9 $$Bias = \frac{{P}_{C,FEM}}{{P}_{C,EXP}}$$

**Fig. 9:**
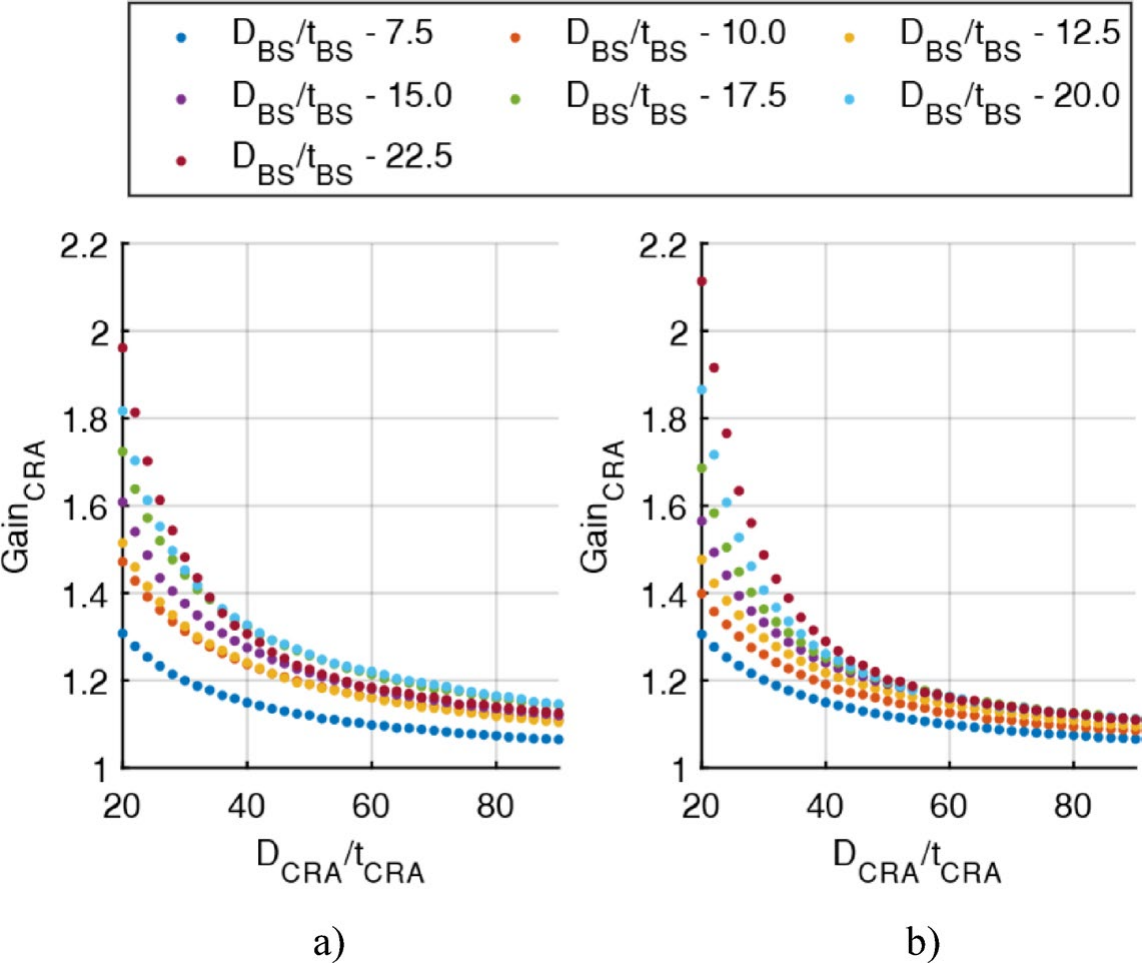
Increase in collapse resistance due to the presence of the liner as a function of the D_CRA_/t_CRA_ ratio. (**a**) 0.1% ovality and µs = 0; (**b**) 1.0% ovality and µs = 0.

The influence of the D_CRA_/t_CRA_ ratio on the collapse pressure can be visualized in Fig. 9a and b, for initial pipes ovalities of 0.1% and 1%, respectively; in these figures, the vertical axis is the gain in collapse resistance due to the liner (Gain_CRA_), calculated according to Eq. 10, where the gain is defined as the ratio of the collapse resistance of the MLP, P_C,MLP_, to the collapse resistance of the backing steel, P_C,BS_. Increasing the D_CRA_/t_CRA_ ratio results in a decrease in the gain in collapse resistance, although this relationship is not linear over the entire analyzed interval. For D_CRA_/t_CRA_ values between 20 and 40, a sharp drop in the gain in collapse resistance is observed for all D_BS_/t_BS_ ratios. As the D_CRA_/t_CRA_ ratio continues to increase, the curves begin to stabilize, presenting a gentler slope. For D_CRA_/t_CRA_ values greater than 60, the curves exhibit asymptotic behavior, converging to the backing steel response.

10 $${Gain}_{CRA} = \frac{{P}_{C,MLP}}{{P}_{C,BS}}$$

**Fig. 10:**
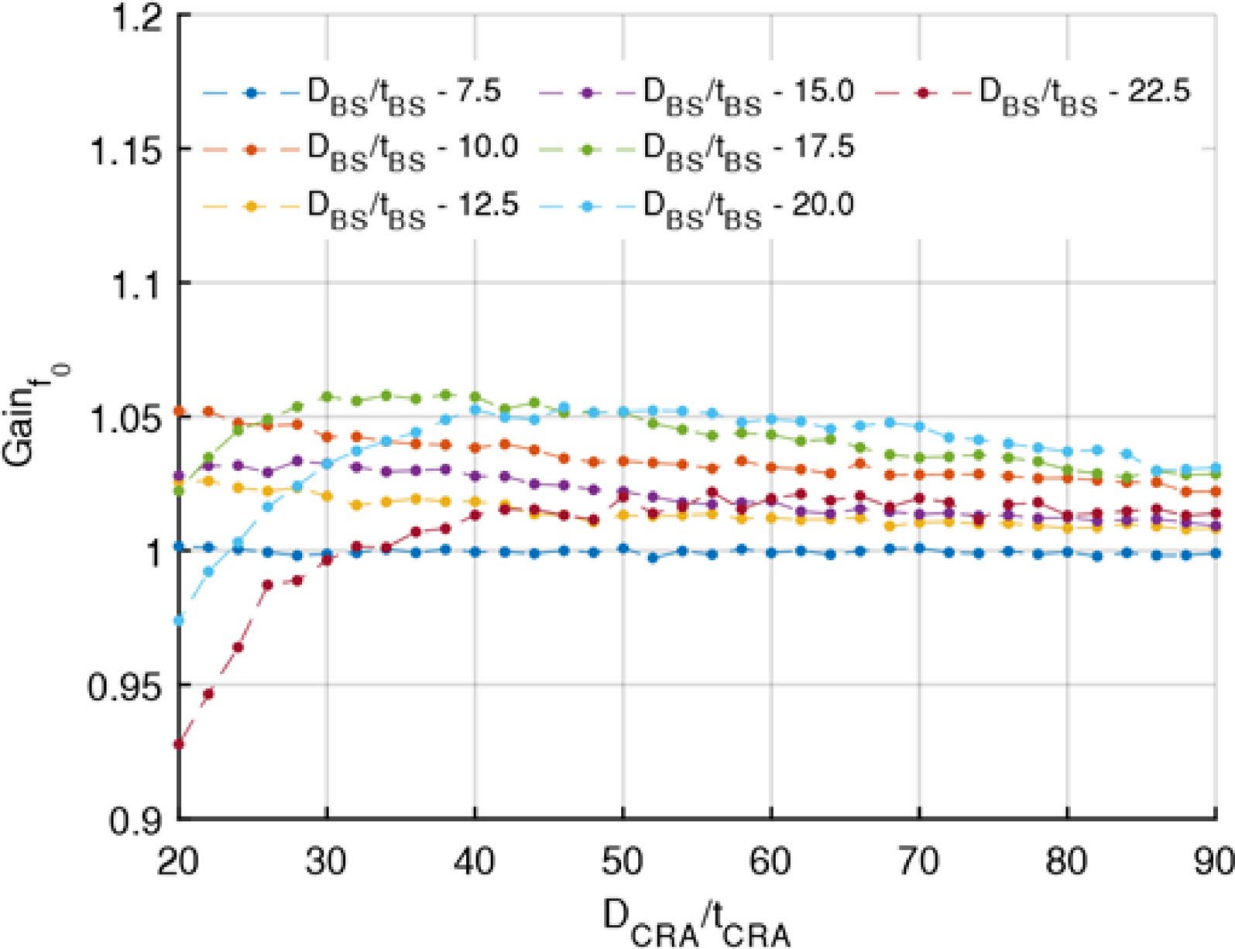
The ratio of liner-induced collapse resistance gains for pipes with 0.1% and 1.0% ovality (Eq. 11), as a function of D_CRA_/t_CRA_ (µ_S_ = 0).

Figure 10 presents the ratio of collapse resistance gains between pipes with 0.1% and 1.0% initial ovality to illustrate how the influence of ovality varies with geometry. The parameter Gain_*f0*_ (Eq. 11) expresses the gain in collapse pressure due to the presence of the liner as ovality decreases in the pipe. The curves in Fig. 10 show that the influence of ovality on collapse resistance changes substantially as the backing‑steel slenderness increases. For low D_BS_/t_BS_ values, the difference between the 0.1% and 1.0% ovality cases remains small and relatively constant across all liner configurations, indicating that collapse is governed primarily by material yielding and is therefore less sensitive to geometric imperfections. As D_BS_/t_BS_ increases, however, the curves show that slender pipes experience a larger reduction in collapse pressure when ovality increases from 0.1% to 1.0%. This widening gap signals the onset of geometric‑instability‑dominated behavior, in which even modest ovality becomes a controlling parameter.

The role of the liner, expressed through the D_CRA_/t_CRA_ ratio, acts on top of this shift. When the liner is thin (high D_CRA_/t_CRA_), its ability to suppress ovalization is limited, and the sensitivity to ovality remains pronounced—especially in pipes with high D_BS_/t_BS_. Under these conditions, the difference between the two ovality levels is amplified, driving larger Gain_*f0*_ values and reinforcing the dominance of geometric imperfection as the governing mechanism. As the liner becomes thicker (lower D_CRA_/t_CRA_), it increasingly constrains cross‑sectional deformation, reducing the amplification of geometric imperfections. This additional stiffness causes the collapse pressures associated with 0.1% and 1.0% ovality to converge, particularly in the high‑slenderness region, where the stabilizing of the liner effect is most needed.

Thus, the transitions observed in the Gain_*f0*_ curves at high D_BS_/t_BS_ arise from the competition between two mechanisms: the increasing imperfection‑sensitivity of slender backing steel, which tends to widen the gap between low‑ and high‑ovality collapse pressures, and the growing stabilizing effect of a thicker liner, which reduces this gap by suppressing ovalization. The shape and separation of the curves in Fig. 10 reflect the balance between these opposing trends.

11 $${Gain}_{{f}_{0}} = {\left(\frac{{P}_{C,MLP}}{{P}_{C,BS}}\right)}_{{f}_{0}=\mathrm{0,1}\%}/{\left(\frac{{P}_{C,MLP}}{{P}_{C,BS}}\right)}_{{f}_{0}=\mathrm{1,0}\%}$$

**Fig. 11:**
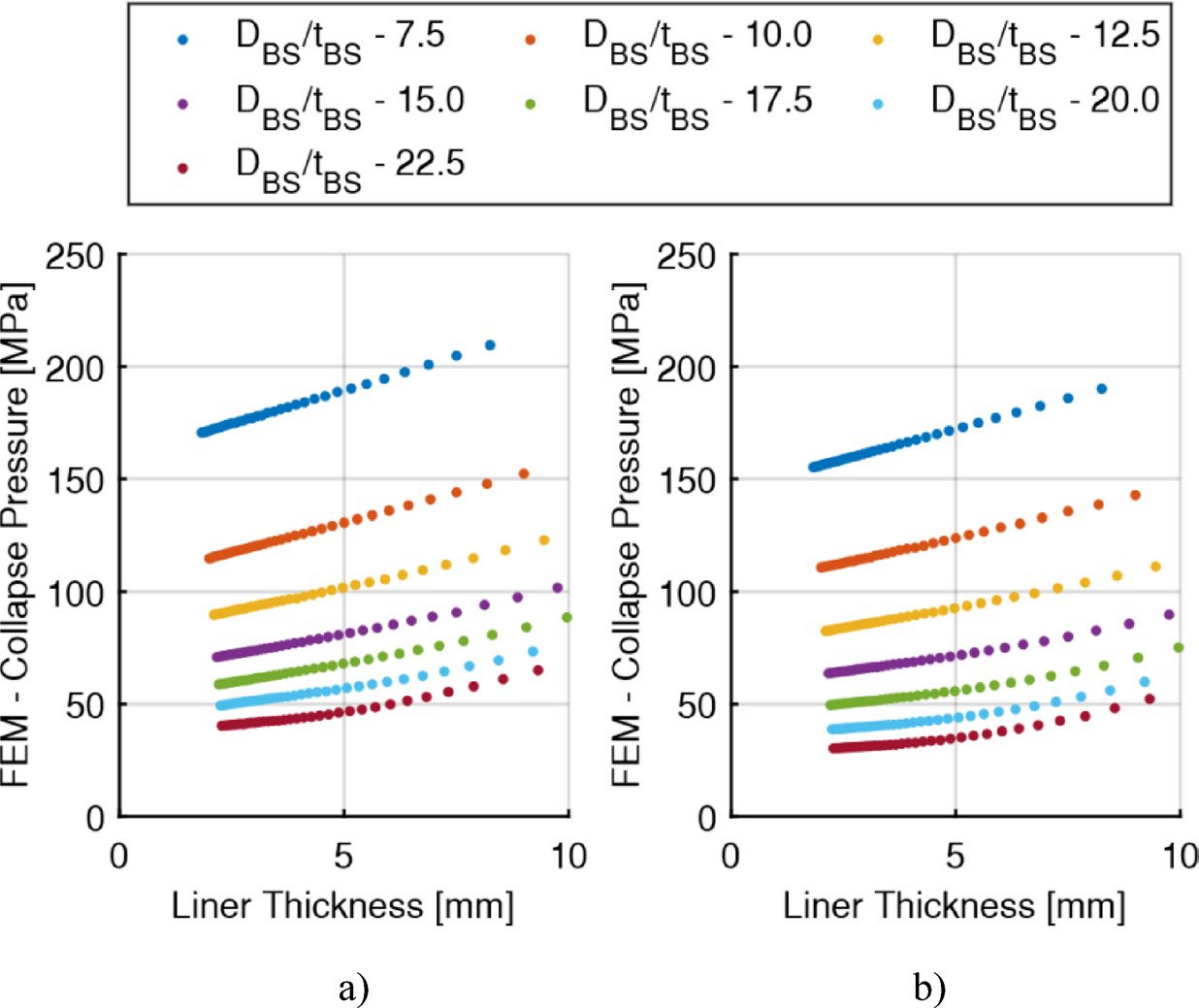
Collapse pressure behavior as a function of CRA liner thickness (µ_S_ = 0). (**a**) 0.1% ovality; (**b**) 1.0% ovality.

Figure 11 illustrates the relationship between CRA liner thickness and collapse pressure across various D_BS_/t_BS_ ratios, based on numerical models that exclude frictional effects. The primary finding is that the liner substantially increases collapse resistance compared to unlined (bare) pipes.

Specifically, for the liner thicknesses evaluated, numerical analyses quantify this increase as 16% to 34% for 3 mm liners applied to pipes with a backing-steel thickness from 9.9 to 24.3 mm, and 22% to 47% for 4 mm liners applied to pipes with a backing-steel thickness from 11.0 to 25.7 mm. Furthermore, the results reveal that the effectiveness of the liner is not uniform; it is most pronounced in more slender configurations (i.e., higher D_BS_/t_BS_ ratios). These slender pipes, having lower inherent structural stiffness, exhibit steeper gains in collapse pressure as liner thickness increases.

**Fig. 12:**
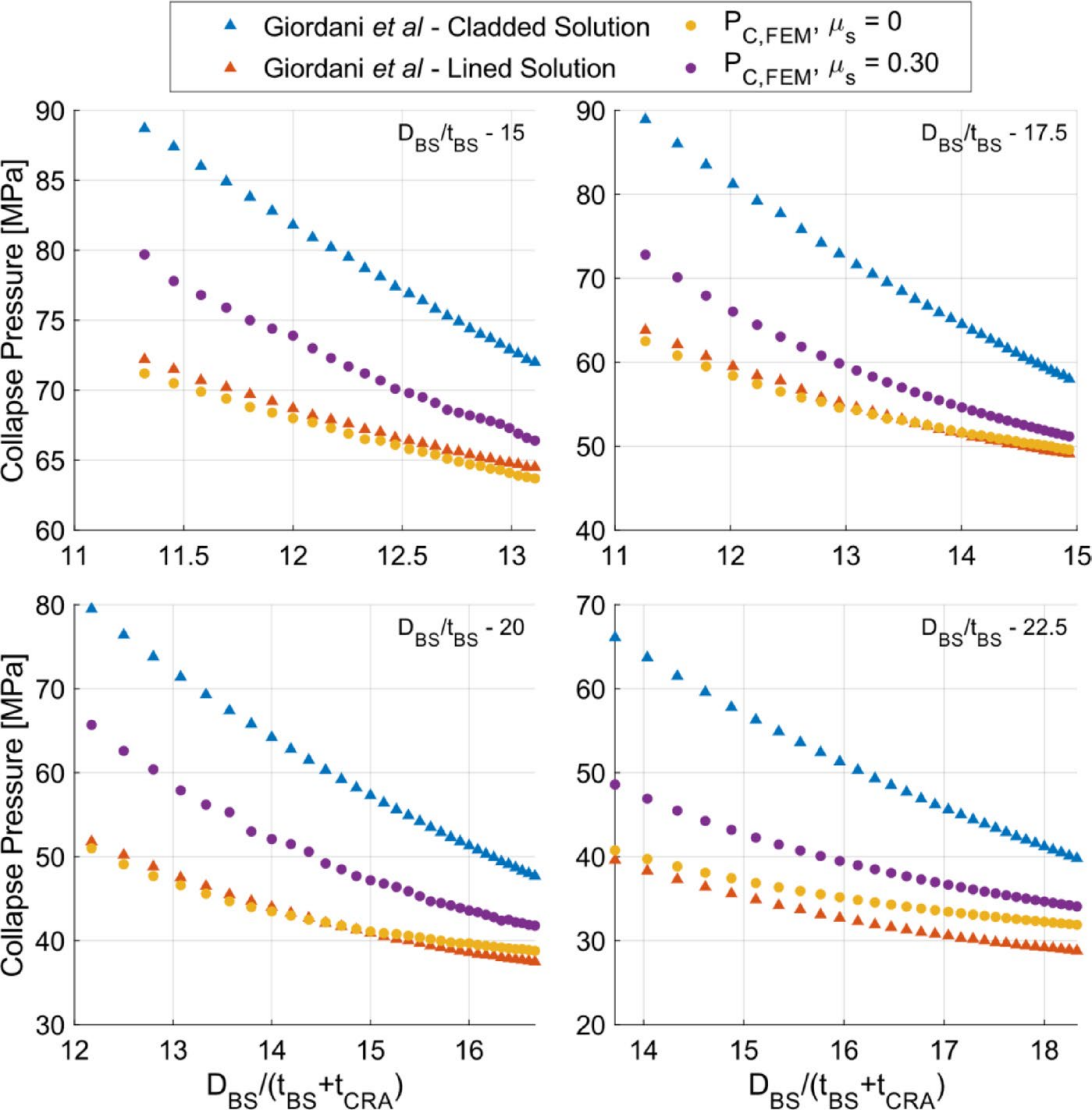
Comparison of collapse pressure values obtained from numerical simulations conducted in the present study and the analytical solution proposed by Giordani et al. (f_0_ = 1.0%), for D_BS_/t_BS_ ratios of 15, 17.5, 20 and 22.5.

Figure 12 shows a comparison between the numerical simulation results from the current study and the analytical values of metallurgically cladded and lined pipes according to *Giordani *et al.^20^. Given the constraints of the analytical solution, this comparison was limited to D_BS_/t_BS_ ratios ranging from 15 to 22.5, and a 1.0% ovality was considered in all cases. The collapse pressure is presented as a function of the D_BS_/t_TOTAL_ ratio, where t_TOTAL_ is defined as the combined t_BS_ + t_CRA_ thickness.

For the cladded case, the analytical solution proposed by Giordani et al*.*^20^ assumes the backing steel and the CRA liner are bonded, whereas for the lined pipe case their solution considers a frictionless interface between the liner and backing steel. The comparison between the analytical solution for lined cases and the frictionless numerical simulations (P_C,FEM_, µ_S_ = 0) in this study shows a satisfactory agreement. The mean difference between the analytical solution and the numerical results was 1.06% for D_BS_/t_BS_  = 15, 0.98% for D_BS_/t_BS_ = 17.5, 1.91% for D_BS_/t_BS_  = 20, and 7.63% for D_BS_/t_BS_  = 22.5.

**Fig. 13:**
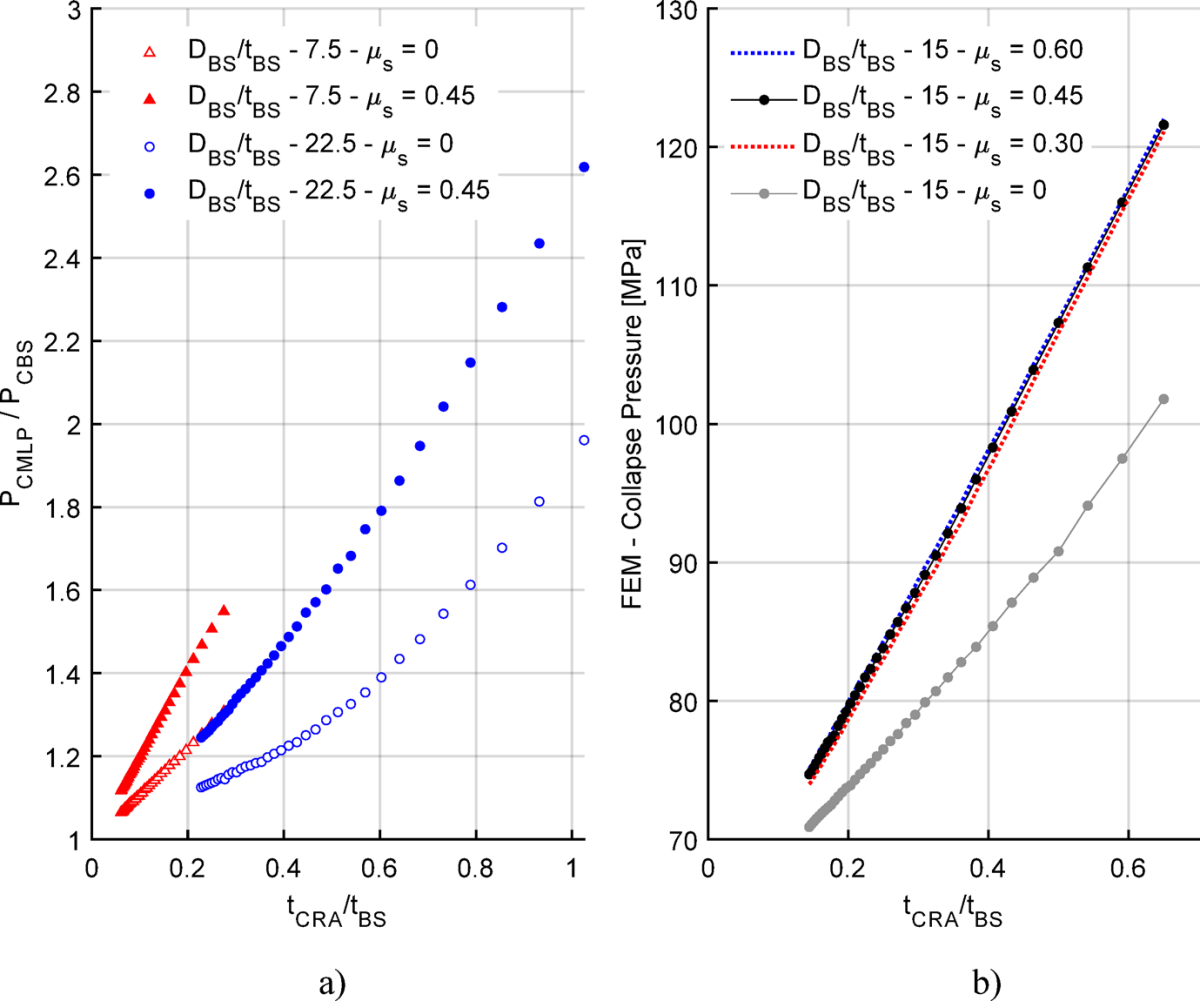
Effects of friction on MLP collapse pressure (f_0_ = 0.1%): (**a**) Collapse resistance gains provided by the liner as a function of the t_CRA_/t_BS_ ratio, comparing high and low D_BS_/t_BS_ ratios under both frictionless and measured friction conditions; (**b**) Influence of the friction coefficient on the collapse pressure for an intermediate D_BS_/t_BS_ ratio, as a function of the t_CRA_/t_BS_ ratio.

The increase in collapse pressure achieved by incorporating the coefficient of friction between the backing steel and the CRA liner is evident when comparing the analytical frictionless lined case with the numerical simulations with friction (P_C,FEM_, µ_S_  = 0.3). This is also clear in Fig. 13a, which shows the combined influence of the mechanical stiffening (caused by increases in liner thickness) and interface friction on the collapse resistance of MLPs. The figure provides a direct comparison between the frictionless condition (µ_s_  = 0) and a case with a friction coefficient of 0.45 in terms of the collapse pressure ratio (P_C,MLP_/P_C,BS_) as a function of the liner-to-pipe thickness ratio (t_CRA_/t_BS_). Two distinct geometries are analyzed: a thick-walled pipe (D_BS_/t_BS_ = 7.5) and a thin-walled pipe (D_BS_/t_BS_  = 22.5), both with 0.1% ovality.

Figure 13b illustrates the sensitivity of collapse pressure to the static friction coefficient, considering the frictionless condition (µ_s_ = 0), the approximate lower and upper bounds of the experimental measurements (µ_s_ = 0.37 and µ_s_ = 0.56), and the mean value (µ_s_ = 0.45), for a reference geometry with D_BS_/t_BS_ = 15. The introduction of friction produces a marked increase in collapse pressure, confirming the immediate onset of composite action between the liner and the backing steel. However, when comparing the simulations for µ_s_ = 0.30, 0.45, and 0.60, the resulting curves are nearly indistinguishable over the entire range of liner thicknesses evaluated. This indicates that µ_s_ = 0.45 is a representative and robust parameter choice, lying within a region where the model exhibits low sensitivity to friction variations. As a consequence, realistic experimental fluctuations in the friction coefficient within the interval 0.30 < µ_s_ < 0.60 do not significantly influence the predicted collapse response, reinforcing the reliability of the numerical results presented.

There is a clear gain in collapse resistance when friction is considered in both pipe geometries. Furthermore, the influence of friction is more pronounced in slender pipes (higher D_BS_/t_BS_ ratios), where the CRA liner becomes critical in maintaining structural integrity. For these geometries, structural stability relies heavily on the ability to transfer shear forces through friction to ensure composite action. In contrast, for thick-walled pipes (lower D_BS_/t_BS_ values), the greater inherent stiffness of the backing steel reduces the relative importance of the frictional contribution of the liner. Even so, the absolute gain from friction remains substantial, highlighting that composite action is a critical mechanism in plasticity-dominated regimes as well.

These results highlight the critical role of interface friction in enhancing the structural performance of MLPs across different geometries. The premise that interfacial friction increases structural stability and collapse resistance of mechanically lined pipes (MLPs) under external pressure is supported by numerical and experimental studies on multilayer tubular mechanics^35,36^. The integrity of the liner depends on maintaining contact with the backing steel pipe, where friction and residual pressure prevent debonding and local collapse, restricting sliding and relative ovalization between layers^36–38^. High friction coefficients delay the formation of collapse lobes, enabling support of high hydrostatic loads, and reduce deformation propagation, making the system behave as a single composite section^10^. Experiments measure the friction coefficient between layers and attempt to validate FEM models, showing significant collapse gains for high D_BS_/t_BS_ ratios when friction is present^36,39^.

## Conclusions

Full-scale collapse tests demonstrated that integrating a CRA liner into a conventional carbon steel pipe enhances resistance to external overpressure under collapse loading conditions. In the tested configurations, this reinforcement led to increases in collapse pressure of up to 43%. These improvements are influenced by several geometric parameters of the mechanically lined pipe (MLP), including liner thickness, the D_BS_/t_BS_ ratio, ovality, and the static friction coefficient.

For specimens with liner thicknesses of 3 mm and 4 mm, greater reinforcement was observed in configurations with higher D_BS_/t_BS_ ratios, resulting in collapse pressure gains approaching 40%. In contrast, for lower D_BS_/t_BS_ ratios (e.g., 8 to 10), the improvement was more modest, around 21%.

However, it is important to note that collapse performance is not governed by the D_BS_/t_BS_ ratio alone. The results suggest a more complex interaction among various parameters, including material properties, interface behaviour, and liner geometry.

Three-dimensional finite element method (3D-FEM) simulations closely matched the experimental collapse pressure results, with deviations within ± 0.1 bias, and a Pearson correlation coefficient of 0.9845. The study further demonstrated that simplified numerical models—constructed using averaged values for geometry, material properties, and interfacial friction—can provide collapse predictions that are in reasonable agreement with those obtained from more detailed simulations incorporating specimen-specific data, achieving a Pearson correlation coefficient of 0.9926.

Among the parameters evaluated, friction and the D_CRA_/t_CRA_ ratio emerged as critical factors in developing a reliable design equation for MLPs. The contribution of the friction coefficient to the collapse resistance is significant, exhibiting a complex, non-linear relationship with the D_BS_/t_BS_ ratio. For instance, as suggested by the numerical results, the reinforcement gain at lower D_BS_/t_BS_ ratios is highly sensitive to the liner-to-backing steel thickness ratio, while configurations with higher D_BS_/t_BS_ ratios show a different non-linear behavior.

The complex interdependencies observed between geometric characteristics, material properties, and interface conditions point to the necessity for multi-variable design approaches, while also highlighting the limitations of simplified equations that treat these effects in isolation. A summary of these findings is presented in Table 2 as a guide for MLP design.

**Table 2:** Design-oriented summary of parametric findings.

Parameter (x)	Value/range	Influence on collapse resistance
D_BS_/t_BS_	x ≥ 20	The thickness of the CRA dominates as a factor of structural reinforcement (gains of up to 40%). The transfer of shear forces between layers through friction is critical for structural stability
	x ≤ 10	The reinforcement provided by the liner is smaller (~ 20%), and less sensitive to the t_CRA_ to t_BS_ ratio. Friction is less influential due to the stiffness of the thick-walled BS pipe
	10 < x < 20	Combinations of D_BS_/t_BS_ and D_CRA_/t_CRA_ can produce similar effects on strength, allowing for a range of customized design configurations
D_CRA_/t_CRA_	x ≥ 50	Higher values lead to approximately linear resistance gains, limited to 20%
	x ≤ 50	Lower ratios (smaller BS wall thickness) lead to non‑linear structural strength gains that exceed 20%
µs	x ≥ 0.45	Strength is higher than in the μ = 0 case for any combination of D_CRA_/t_CRA_ and D_BS_/t_BS_

It must be emphasized that the DNV-ST-F101 collapse expressions, while useful as a baseline reference, do not fully capture the combined effects of CRA liner stiffness, interface friction, and geometric interaction that govern the collapse behavior of MLPs.

Future work in this area should ideally explore the contribution of residual stresses to the gripping force between backing steel and liner, the role of eccentricity combined with the variables discussed in this paper, and the effect of the typical sequences of loading conditions imposed on pipes from manufacturing to in-field installation (i.e. reeling, unreeling and straightening, welding, laying).

## Supplementary Information

Below is the link to the electronic supplementary material.

Supplementary Material 1

## Data Availability

All data supporting the findings of this study are available within the paper and its Supplementary Information.
