# Supplementary material for: Numerical and experimental evaluation of the collapse resistance of MLPs under external pressure by lateral loading
Source: Sci Rep. 2026 Apr 28;16:19740. doi: 10.1038/s41598-026-47504-8 (PMC13316104; doi:10.1038/s41598-026-47504-8)
Supplement: Supplementary file 1 — Supplementary Material 1 [file 41598_2026_47504_MOESM1_ESM.docx]

Supplementary Information

Supplementary Table S1 - Measured diameter-to-thickness ratio (D_BS_/t_BS_), outer diameter (D_BS_), backing steel thickness (t_BS_), liner thickness (t_CRA_), ovality (f_0_), eccentricity (E_CC_), static friction coefficient (µ_S_), backing steel yield strength (Sy _0.5%_ BS), liner yield strength (Sy _0.5%_ CRA), backing steel ultimate strength (S_U_ BS), liner ultimate strength (S_U_ CRA), full-scale testing collapse pressure (P_C_ EXP), collapse pressure DNV (P_C_ DNV), simulated collapse pressure values (P_C_ FEM) and collapse shape.

| Specimen | D_BS_/t_BS_  [-] | D_BS_ [mm] | t_BS_ [mm] | t_CRA_ [mm] | f_0_  [%] | E_CC_  [%] | µ_S_ [-] | P_c_ EXP [MPa] | P_c_ DNV [MPa] | P_c_ FEM [MPa] | Sy_0.5%_ BS  [MPa] | S_U_ BS [MPa] | Sy_0.5%_ CRA [MPa] | S_U_ CRA [MPa] | Collapse Shape |
| --- | --- | --- | --- | --- | --- | --- | --- | --- | --- | --- | --- | --- | --- | --- | --- |
| CP_01 | 8.9 | 216.0 | 24.3 | 2.98 | 0.09 | 2.3 | 0.50 | 138 | 111 | 149 | 496 | 578 | 519 | 805 | Dog bone |
| CP_02 | 8.9 | 216.0 | 24.3 | 2.98 | 0.04 | 2.2 | 0.50 | 137 | 111 | 155 | 496 | 578 | 519 | 805 | Dog bone |
| CP_03 | 9.6 | 215.2 | 22.6 | 3.49 | 0.30 | 10.2 | 0.42 | 138 | 102 | 132 | 507 | 583 | 564 | 858 | Dog bone |
| CP_04 | 9.8 | 196.7 | 20.2 | 3.32 | 0.04 | 4.9 | 0.48 | 128 | 101 | 138 | 496 | 573 | 470 | 785 | Dog bone |
| CP_05 | 10.7 | 274.0 | 25.7 | 4.05 | 0.09 | 1.5 | 0.37 | 112 | 92 | 121 | 496 | 577 | 434 | 770 | Dog bone |
| CP_06 | 12.1 | 187.4 | 15.5 | 3.33 | 0.04 | 4.9 | 0.56 | 105 | 82 | 109 | 473 | 546 | 484 | 793 | U |
| CP_07 | 13.2 | 197.2 | 15.0 | 3.31 | 0.12 | 8.0 | 0.43 | 94 | 74 | 97 | 518 | 584 | 541 | 840 | Dog bone |
| CP_08 | 13.2 | 262.7 | 20.1 | 4.19 | 0.17 | 3.7 | 0.37 | 90 | 74 | 95 | 496 | 577 | 434 | 770 | Dog bone |
| CP_09 | 13.2 | 197.5 | 15.0 | 3.43 | 0.07 | 7.5 | 0.50 | 96 | 75 | 99 | 526 | 602 | 575 | 867 | U |
| CP_10 | 15.2 | 180.3 | 11.9 | 3.13 | 0.01 | 6.8 | 0.49 | 80 | 65 | 87 | 490 | 555 | 454 | 781 | U |
| CP_11 | 15.3 | 180.3 | 11.9 | 3.13 | 0.02 | 6.5 | 0.49 | 79 | 65 | 85 | 490 | 555 | 454 | 781 | U |
| CP_12 | 15.8 | 242.0 | 15.6 | 4.20 | 0.25 | 5.2 | 0.40 | 84 | 60 | 78 | 499 | 577 | 424 | 772 | U |
| CP_13 | 16.2 | 178.3 | 11.0 | 3.13 | 0.01 | 7.8 | 0.43 | 79 | 61 | 82 | 488 | 567 | 465 | 775 | U |
| CP_14 | 17 | 178.2 | 10.6 | 3.14 | 0.01 | 9.3 | 0.43 | 73 | 58 | 77 | 488 | 567 | 465 | 775 | U |
| CP_15 | 17.1 | 177.2 | 10.5 | 3.13 | 0.02 | 6.5 | 0.49 | 73 | 58 | 76 | 498 | 576 | 462 | 778 | U |
| CP_16 | 17.8 | 177.2 | 10.0 | 3.09 | 0.05 | 7.3 | 0.49 | 71 | 55 | 71 | 498 | 576 | 462 | 778 | Dog bone |
| CP_17 | 17.9 | 176.0 | 9.9 | 3.10 | 0.05 | 11.7 | 0.48 | 61 | 55 | 71 | 496 | 573 | 470 | 785 | U |
| CP_18 | 18.2 | 250.1 | 13.9 | 4.00 | 0.06 | 5.8 | 0.37 | 67 | 54 | 66 | 496 | 577 | 434 | 770 | Dog bone |
| CP_19 | 19.7 | 234.2 | 12.1 | 4.23 | 0.19 | 5.8 | 0.40 | 66 | 46 | 60 | 499 | 577 | 424 | 772 | Dog bone |
| CP_20 | 20.8 | 228.0 | 11.0 | 3.33 | 0.06 | 11.0 | 0.40 | 55 | 45 | 55 | 508 | 584 | 487 | 821 | U |
| CP_21 | 21.2 | 232.1 | 11.0 | 4.19 | 0.07 | 6.9 | 0.40 | 59 | 43 | 57 | 499 | 577 | 424 | 772 | Dog bone |

Supplementary Table S2 – Mesh convergence study results. n_Lenght_ is the number of elements in the longitudinal direction; n_Thickness,BS_ and n_Thickness,CRA_ are the number of elements through the thickness of the backing steel and the CRA liner, respectively; n_Circunf_ is the number of elements in the circumferential direction.

| Mesh Case | n_Length_ | n_Thickness,BS_ | n_Thickness,CRA_ | n_Circumf_ | Total Elements | P_C_ [MPa] | Variation [%] |
| --- | --- | --- | --- | --- | --- | --- | --- |
| 1 | 20 | 2 | 2 | 20 | 27.513 | 261.3 | - |
| 2 | 30 | 3 | 3 | 25 | 29.081 | 267.1 | 2.22 |
| 3 | 40 | 4 | 4 | 30 | 31.188 | 271.5 | 1.65 |
| 4 | 45 | 5 | 5 | 35 | 35.701 | 274.3 | 1.03 |
| **5** | **45** | **6** | **6** | **40** | **39.968** | **274.6** | **0.11** |
| 6 | 50 | 7 | 7 | 45 | 46.116 | 274.7 | 0.04 |

A mesh convergence study was performed by progressively refining the mesh and evaluating the corresponding collapse pressure (P_C_). In this process, the number of elements in the thickness, circumferential, and longitudinal directions was increased simultaneously, maintaining a consistent aspect ratio between elements and avoiding highly distorted meshes.

Although isolating each discretization direction could provide a more detailed assessment of their individual influence, this approach was not adopted in the present study. This decision was made to ensure that the mesh refinement remained representative of the actual numerical model used in the simulations, where all directions are inherently coupled.

The results presented on Table S2 showed a clear convergence trend, with the variation in collapse pressure becoming smaller than 1% for meshes with approximately 40.000 elements. Based on this criterion, the selected mesh represents an adequate compromise between numerical accuracy and computational cost.
